# Blockade of TRPM7 Channel Activity and Cell Death by Inhibitors of 5-Lipoxygenase

**DOI:** 10.1371/journal.pone.0011161

**Published:** 2010-06-17

**Authors:** Hsiang-Chin Chen, Jia Xie, Zheng Zhang, Li-Ting Su, Lixia Yue, Loren W. Runnels

**Affiliations:** 1 Department of Pharmacology, Robert Wood Johnson Medical School, University of Medicine and Dentistry of New Jersey, Piscataway, New Jersey, United States of America; 2 Calhoun Cardiology Center and Department of Cell Biology, University of Connecticut Health Center, Farmington, Connecticut, United States of America; INSERM U1016, Institut Cochin, France

## Abstract

TRPM7 is a ubiquitous divalent-selective ion channel with its own kinase domain. Recent studies have shown that suppression of TRPM7 protein expression by RNA interference increases resistance to ischemia-induced neuronal cell death *in vivo* and *in vitro*, making the channel a potentially attractive pharmacological target for molecular intervention. Here, we report the identification of the 5-lipoxygenase inhibitors, NDGA, AA861, and MK886, as potent blockers of the TRPM7 channel. Using a cell-based assay, application of these compounds prevented cell rounding caused by overexpression of TRPM7 in HEK-293 cells, whereas inhibitors of 12-lipoxygenase and 15-lipoxygenase did not prevent the change in cell morphology. Application of the 5-lipoxygenase inhibitors blocked heterologously expressed TRPM7 whole-cell currents without affecting the protein's expression level or its cell surface concentration. All three inhibitors were also effective in blocking the native TRPM7 current in HEK-293 cells. However, two other 5-lipoxygenase specific inhibitors, 5,6-dehydro-arachidonic acid and zileuton, were ineffective in suppressing TRPM7 channel activity. Targeted knockdown of 5-lipoxygenase did not reduce TRPM7 whole-cell currents. In addition, application of 5-hydroperoxyeicosatetraenoic acid (5-HPETE), the product of 5-lipoxygenase, or 5-HPETE's downstream metabolites, leukotriene B4 and leukotriene D4, did not stimulate TRPM7 channel activity. These data suggested that NDGA, AA861, and MK886 reduced the TRPM7 channel activity independent of their effect on 5-lipoxygenase activity. Application of AA861 and NDGA reduced cell death for cells overexpressing TRPM7 cultured in low extracellular divalent cations. Moreover, treatment of HEK-293 cells with AA861 increased cell resistance to apoptotic stimuli to a level similar to that obtained for cells in which TRPM7 was knocked down by RNA interference. In conclusion, NDGA, AA861, and MK886 are potent blockers of the TRPM7 channel capable of attenuating TRPM7's function during cell stress, making them effective tools for the biophysical characterization and suppression of TRPM7 channel conductance *in vivo*.

## Introduction

TRPM7, a member of the transient receptor potential melastatin-like (TRPM) ion channel subfamily, is a widely expressed bifunctional protein with both ion channel and α-kinase domains [1], [2]. Several physiological functions have been ascribed to the channel-kinase including magnesium and rare metal homeostasis, melanopore maturation, kidney stone formation, sensing of sheer stress, synaptic vesicle fusion, thymopoiesis, and cell adhesion [3], [4], [5], [6], [7], [8], [9], [10]. In addition, the channel also contributes to ischemic brain pathology. It has been shown that TRPM7 channel activity is up-regulated in oxygen glucose deprived cortical neurons and that knockdown of TRPM7 expression by RNA interference in cultured neurons and the hippocampus delayed anoxic cell death [11], [12].

Several modulators of TRPM7 channel activity, including PIP_2_, Mg^2+^, Mg^2+^•ATP, polyvalent cations, and H^+^ have been identified [1], [13], [14], [15], [16]. The phospholipid PIP_2_ gates the channel such that activation of receptors coupled to phospholipase C (PLC) inhibits channel activity by depleting cellular PIP_2_ [15], [17]. Mg^2+^, Mg^2+^•ATP, H^+^, and polyvalent cations are believed to inhibit channel activity by screening PIP_2_ head group charges [13]. Thus, the prevailing view is that in unstimulated cells a large fraction of TRPM7's channel activity is suppressed by Mg^2+^, Mg^2+^•ATP, and other factors, and that activation of the channel is achieved by reversing the action of these modulators on the channel [18].

To better understand the mechanism(s) by which the channel contributes to the demise of cells under cellular stress, we characterized the cellular effects produced by increased TRPM7 channel activity employing HEK-293 cells as a model [10]. Overexpression of the channel-kinase in HEK-293 cells produced cell rounding that was dependent upon the calcium-dependent protease m-calpain [10]. More recently, we have shown that TRPM7 activates m-calpain through reactive oxygen species (ROS) dependent activation of the stress-activated protein kinases p38 MAP kinase and c-Jun N-terminal kinase (JNK) in HEK-293 cells [19]. During the course of our investigation into the mechanism by which overexpression of TRPM7 caused loss of cell adhesion, we discovered that application of the non-specific lipoxygenase inhibitor NDGA attenuated cell rounding and loss of adhesion produced by influx of divalent cations through the channel. Electrophysiological measurements demonstrated that application of NDGA potently inhibited TRPM7 channel activity, suggesting that a lipoxygenase (LOX) may be involved in regulating the channel. Lipoxygenases are a family of calcium-dependent dioxygenases, including 5-lipoxygenase (5-LOX), 12-lipoxygenase (12-LOX), and 15-lipoxygenase (15-LOX), that metabolize arachidonic acid to distinct biologically active fatty acid hydroperoxides, such as leukotrienes and hydroyeicosateraenoic acids [20]. These metabolites take part in numerous cell processes, including inflammation, proliferation, cell invasion, angiogenesis, cell adhesion, and cell spreading [21], [22], [23], [24]. It is well known that lipid molecules modulate several members of the TRP family [25]. In particular, products of lipoxygenase have been demonstrated to directly modulate TRPV1 channel activity [26]. Activation of histamine and bradykinin receptors stimulates TRPV1 channel activity in a 12-LOX-dependent manner [27], [28]. Thus, our discovery that NDGA blocked TRPM7 channel activity initially suggested to us that TRPM7 channel activity may be similarly controlled by a lipoxygenase. However, results from our experiments indicate that these compounds block TRPM7 channel activity independent of their actions on 5-LOX. Here we identify the 5-LOX inhibitors NDGA, AA861, and MK886 as potent blockers of the TRPM7 channel and demonstrate that depletion of TRPM7 channel activity by RNA interference or by treatment of cells with TRPM7 channel blockers reduces cell death caused by apoptotic stimuli.

## Results

**Figure 1:**
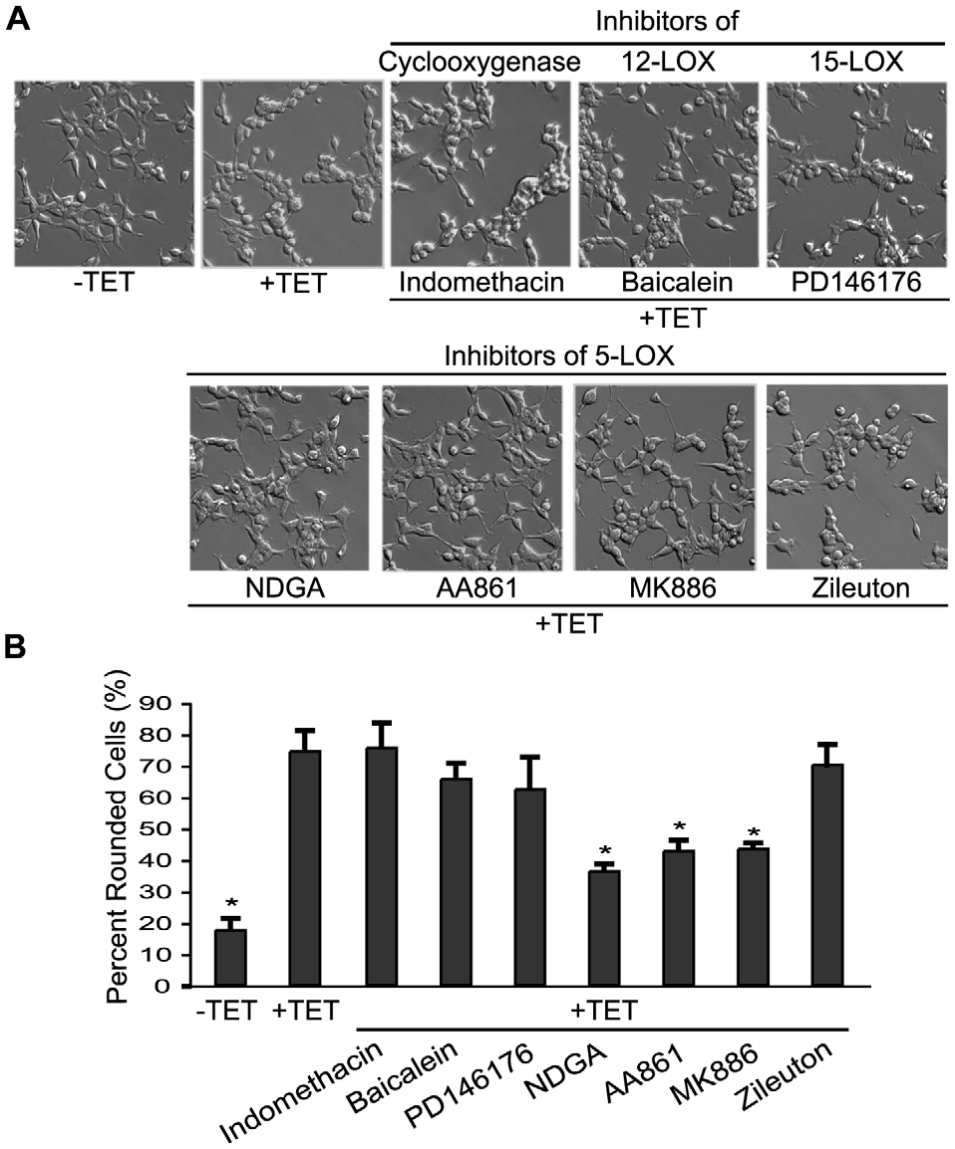
Inhibitors of 5-LOX block TRPM7-mediated cell rounding. (A) Application of the 5-LOX inhibitors NDGA (10 µM), AA861 (10 µM), and MK886 (10 µM) to 293-TRPM7 expressing cells reduced cell rounding. In contrast, treatment of 293-TRPM7 cells with the cyclooxygenase inhibitor indomethacin (10 µM), 12-LOX inhibitor baicalein (5 µM), 15-LOX inhibitor PD146176 (2 µM), and the 5-LOX inhibitor zileuton (50 µM) did not block TRPM7-induced cell rounding. (B) Quantification of the degree of cell rounding under the conditions depicted in (A). Values are mean ± standard deviation of at least three independent experiments. A χ^2^ test was employed to assess differences in cell rounding between 293-TRPM7 cells treated with the different inhibitors. An asterisk indicates treatments that produced a decrease in cell rounding that was significantly different from 293-TRPM7 cells grown in tetracycline.

Previously, we have shown that overexpression of TRPM7 in HEK-293 cells caused cell rounding and loss of adhesion that occurred in a channel-dependent and kinase-independent manner [10]. Cell rounding induced by TRPM7 could be prevented by pharmacological inhibition of calpain as well as by reduction of m-calpain protein levels by RNA interference. An investigation into the mechanism by which TRPM7 controls m-calpain revealed that application of NDGA, an inhibitor that is effective against 5-, 12- and 15-LOX, inhibited TRPM7-dependent cell rounding with a measured IC_50_ (half maximal inhibitory concentration) of 6.3 µM (Fig. 1A,B) [29], [30]. To further investigate a potential role for a lipoxygenase in controlling TRPM7 channel function, we employed a cell rounding assay with the 293-TRPM7 cell line, which overexpresses TRPM7 in HEK-293 cells in a tetracycline-inducible manner [10]. Inhibitors of 12-LOX (baicalein) and 15-LOX (PD146176) were ineffective in blocking TRPM7-dependent cell rounding (Fig. 1A,B). In addition, indomethacin, an inhibitor of cyclooxygenases (an enzyme that also uses arachidonic acid as a substrate), did not block cell rounding. However, the 5-LOX inhibitors AA861 and MK886, which binds to and inhibits 5-lipoxygenase-activating-protein (FLAP), suppressed TRPM7-induced cell rounding with measured IC_50_s of 6.0 and 8.6 µM, respectively (Fig. 1A,B) [21]. The oral 5-LOX inhibitor zileuton, however, was not effective in blocking cell rounding (Fig. 1A, B).

**Figure 2:**
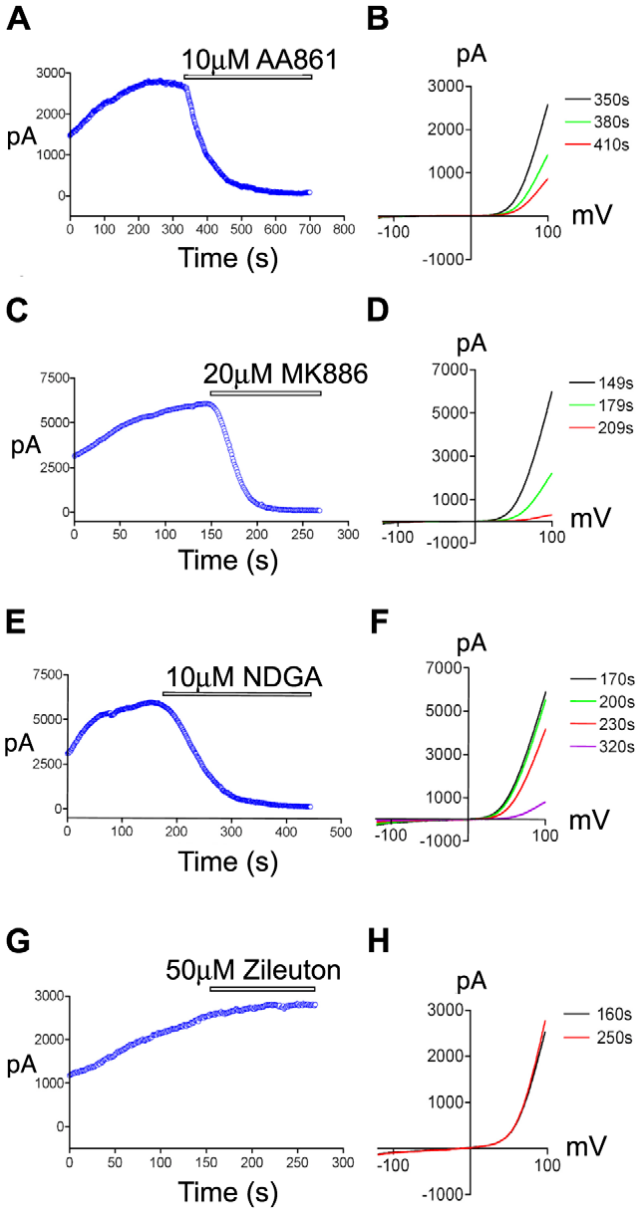
The effects of 5-LOX inhibitors on TRPM7 channel activity. Application of the 5-LOX inhibitors AA861 (A), MK886 (C), and NDGA (E) to 293-TRPM7 expressing cells decreased the heterologously expressed TRPM7 current amplitude over time (+100 mV). Representative traces showing the TRPM7 current-voltage relationship before and after application of AA861 (B), MK886 (D), and NDGA (F). In contrast, the 5-LOX inhibitor, zileuton, did not alter the TRPM7 current amplitude over time (G) or its current-voltage relationship (H). The above experiments were performed a minimum of 10 times with similar results.

To assess the ability of the 5-LOX inhibitors to block TRPM7 channel activity, we next performed whole-cell recordings of TRPM7 current from cells overexpressing the channel-kinase (Fig. 2A-F). NDGA and AA861 completely blocked TRPM7 channel activity at the concentrations used to inhibit cell rounding (Fig. 2A,B,E,F), with higher concentrations of the compounds blocking more rapidly (Fig. S1). We were unable to measure the effect of MK886 on channel conductance at lower concentrations due to the compound's slow onset of inhibition and tendency to destroy the seal and increase leak current. However, higher concentrations of the compound were very effective in blocking TRPM7 channel activity (Fig. 2C,D). Consistent with zileuton's lack of effect on TRPM7-induced cell rounding, the 5-LOX inhibitor had no effect on whole-cell currents (Fig. 2 G,H), leaving it unclear whether 5-LOX was playing an important role in controlling TRPM7 currents.

**Figure 3:**
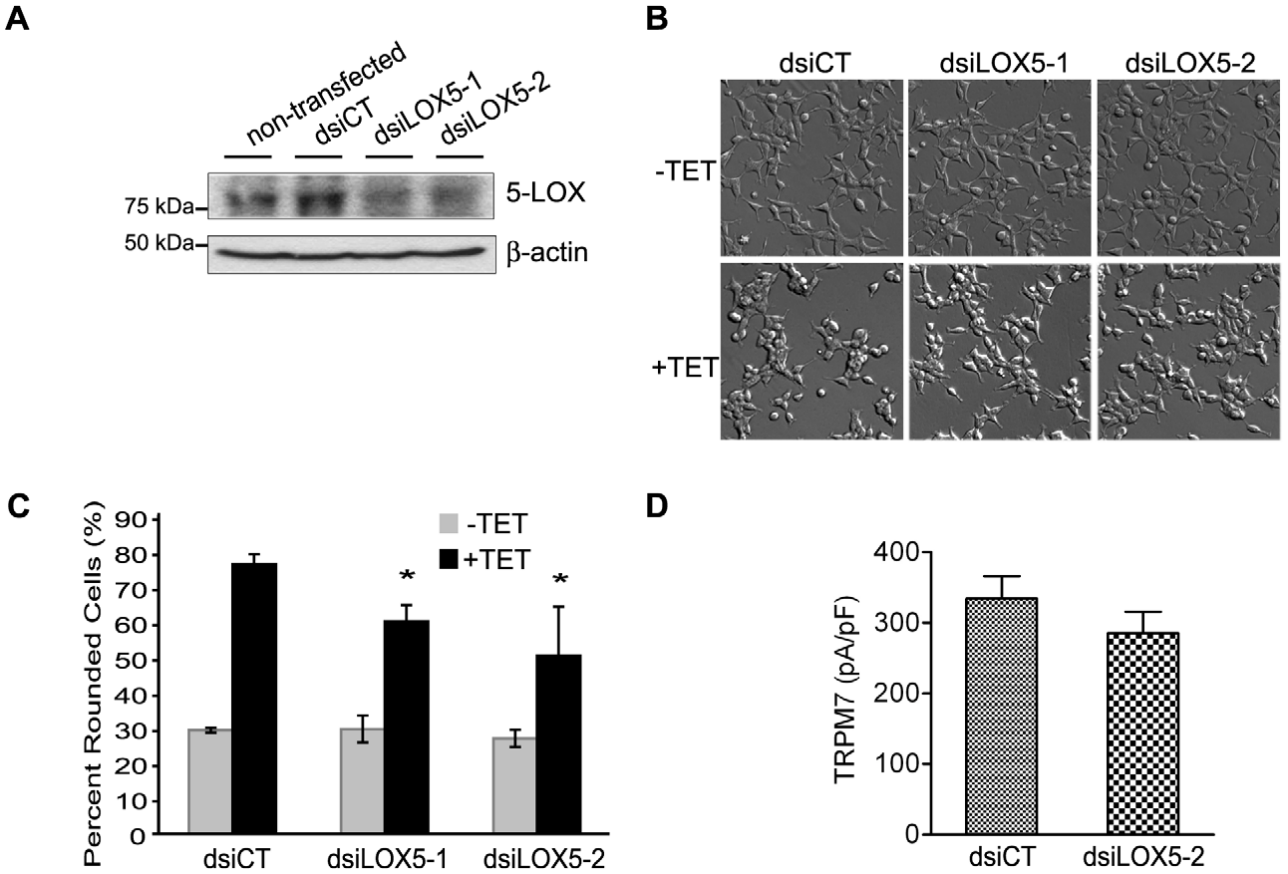
The effect of 5-LOX on TRPM7-mediated cell rounding and TRPM7 current density. (A) A western blot using an antibody against 5-LOX revealed the efficiency of the dsiRNA-mediated knockdown of endogenous 5-LOX in 293-TRPM7 cells. 293-TRPM7 cells were transfected with 10 nM dsiRNAs targeting 5-LOX (dsiLOX5-1 & dsiLOX5-2) and the non-silencing control dsiRNA (dsiCT). Cells were lysed 48 hours after transfection. A western blot of β-actin is shown to demonstrate the equal loading of samples. (B) Transfection of dsiRNAs targeting 5-LOX modestly decreased TRPM7-mediated cell rounding. (C) Quantification of the degree of cell rounding under the conditions depicted in (B). Values are presented as the mean ± standard deviation of three independent experiments. The asterisk indicates a significant difference in cell rounding between 5-LOX knockdown (dsiLOX5-1 & dsiLOX5-2) and control cells (dsiCT), using Student's *t* test (*p*<0.05) (D) Knockdown of 5-LOX did not appreciably decrease TRPM7's current density at +100 mV in 293-TRPM7 expressing cells transfected with dsiLOX5-2 compared to cells transfected with the control (dsiCT). *p* = 0.2869 was compared with control cells (dsiCT) using Student's *t* test (*p*>0.1).

To investigate whether 5-LOX was involved in the regulation of TRPM7 channel activity, two Dicer substrate small interfering RNAs (dsiRNAs) targeting human 5-lipoxygenase (dsiLOX5-1 and dsiLOX5-2) were designed and characterized for their ability to modulate 5-LOX expression. Transfection of dsiLOX5-1 and dsiLOX5-2 suppressed expression of heterologously expressed GFP-5-LOX by 53% and 67%, respectively, without affecting expression of heterologously expressed GFP-15-LOX-2 (Fig. S2A,B). Transfection of dsiLOX5-1 and dsiLOX5-2 into 293-TRPM7 cells each decreased expression of the endogenous 5-lipoxygenase by greater than 50% compared to cells transfected with the non-silencing control dsiRNA (dsiCT) (Fig. 3A). Knockdown of endogenous 5-LOX partially suppressed TRPM7-induced cell rounding (Fig. 3B,C), however, no appreciable differences in whole-cell currents from 293-TRPM7 cells transfected with dsiLOX5-2 compared to cells transfected with the non-silencing control dsiCT were observed (Fig. 3D). This data indicated that knockdown of 5-LOX likely attenuated cell rounding by limiting ROS production and calpain activation, rather than by directly interfering with TRPM7 channel activity [19].

To further determine whether the 5-LOX pathway was involved in the regulation of the TRPM7 channel, we tested whether application of 5-hydroperoxyeicosatetraenoic acid (5-HPETE), the product of 5-LOX, could reactivate TRPM7 whole-cell currents in the presence of AA861. However, neither application of 5-HPETE or its downstream metabolites, leukotriene B4 (LTB4) and leukotriene D4 (LTD4), perfused into the extracellular buffer or added to the internal pipette solution, were able to restore channel activity (Fig. S3). We also investigated the possibility that build-up of arachidonic acid from 5-LOX inhibition was responsible for blocking TRPM7 channel activity. However, application of 10 µM arachidonic acid did not affect whole-cell currents (Fig. S4C,D). Moreover, another 5-LOX specific inhibitor, zileuton, an oral drug for asthma, did not block TRPM7 channel activity within the time-course of our electrophysiological recordings (Fig. 2G,H) [21]. Similar results were obtained for the 5-LOX inhibitor 5,6-dehydro-arachidonic acid (5,6-DAA) (Fig. S4A,B). Another possibility we considered was that 5-LOX could be directly interacting with TRPM7. However, co-immunoprecipitation experiments using cell lysates from cells heterologously expressing TRPM7 and the GFP fusion protein of 5-lipoxygenase (GFP-5-LOX) failed to reveal an interaction between theses two proteins (data not shown). Collectively, these results indicated that NDGA, AA861, and MK886 were blocking TRPM7 channel activity independent of their actions on 5-LOX.

**Figure 4:**
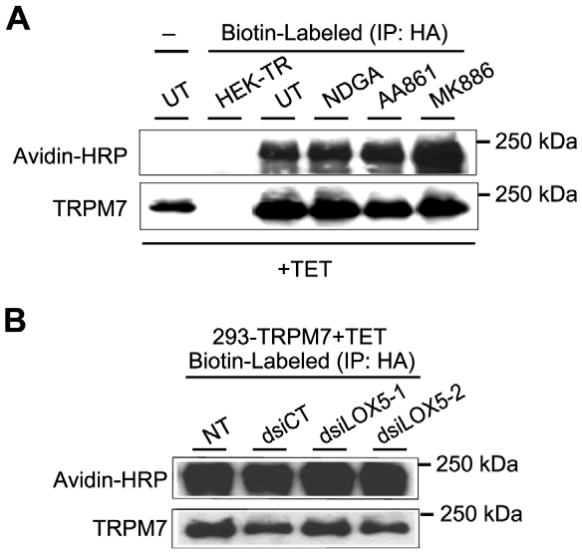
The effect of 5-LOX inhibitors on TRPM7 protein expression and cell surface concentration. (A) 293-TRPM7 expressing cells were treated with NDGA (10 µM), AA861 (10 µM), and MK886 (10 µM) for 24 hours. Control HEK-293 cells not expressing the channel (HEK-TR) as well as 293-TRPM7 cells, treated or not treated with the 5-LOX inhibitors (UT), were subjected to surface labeling with biotin. The cells were lysed and TRPM7 was subsequently immunoprecipitated using a HA antibody and the amount of biotinylated protein was detected by western blotting using Avidin-HRP to detect the biotin label. Untreated cells (UT) that were not biotinylated (-) served as a negative control. Western blotting using a monoclonal antibody against the HA tag indicated that the 5-LOX inhibitors did not appreciably affect TRPM7 expression. In addition, detection of the biotin label indicated that 5-LOX inhibitors did not affect the surface concentration of the channel. The HEK-TR cell line, from which 293-TRPM7 cells were derived, was used as a negative control. (B) Transfection of 293-TRPM7 cells with dsiRNAs against 5-LOX (dsiLOX5-1 & dsiLOX5-2) did not affect TRPM7 surface levels or protein expression compared to non-transfected (NT) cells or cells transfected with the control (dsiCT).

Since the results thus far indicated that NDGA, AA861, and MK886 were not blocking TRPM7 channel activity by interfering with 5-LOX, we next investigated whether these compounds blocked TRPM7 channel activity by interfering with TRPM7 protein expression or with trafficking of the ion channel to the cell surface. However, the expression levels of TRPM7 in 293-TRPM7 cells treated with these 5-LOX inhibitors were unchanged (Fig. 4A). In addition, we were unable to detect any significant difference in cell surface biotinylation of TRPM7 in cells treated with the 5-LOX inhibitors (Fig. 4A) or in cells in which 5-LOX has been knocked down (Fig. 4B). These results indicated that the 5-LOX inhibitors were not blocking TRPM7 channel activity by affecting TRPM7 protein expression or its concentration on the cell surface.

**Figure 5:**
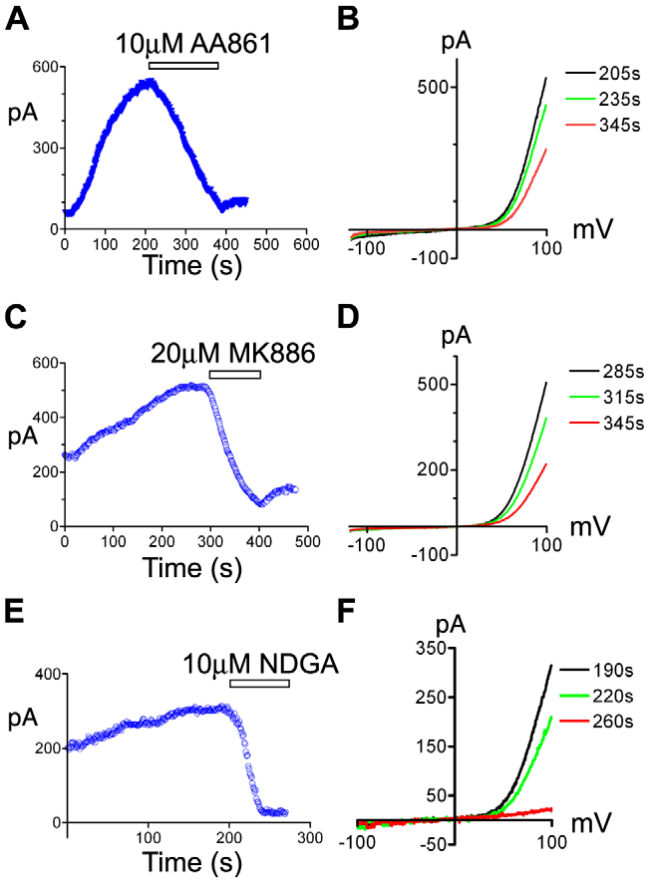
The effects of 5-LOX inhibitors on endogenous TRPM7 channel activity. Application of the 5-LOX inhibitors AA861 (A), MK886 (C), and NDGA (E) decreased the endogenous TRPM7 current amplitude in HEK-293 cells over time (+100 mV). Representative traces showing the TRPM7 current-voltage relationship before and after application of AA861 (B), MK886 (D), and NDGA (F). The above experiments were performed a minimum of 5 times with similar results.

TRPM7 is a ubiquitously expressed and constitutively active divalent cation channel whose basal activity is regulated by intracellular levels of Mg^2+^ and Mg^2+^•ATP [1], [18]. An endogenous TRPM7-like Mg^2+^-nucleotide-regulated metal current (MagNuM) and magnesium-inhibited cation (MIC) channel have been described in multiple cell types including RBL-2H3 cells, human retinoblastoma cells, cardiomycytes, glia, as well as neurons [1], [31], [32], [33], [34], [35]. The TRPM7-like current is also present in HEK-293 cells and we have recently shown that this current is constituted by TRPM7 [10]. All three compounds, NDGA, AA861, and MK886, blocked the endogenous TRPM7-like current in HEK-293 cells (Fig. 5), indicating that these compounds are effective reagents for inhibiting the native TRPM7 channel.

**Figure 6:**
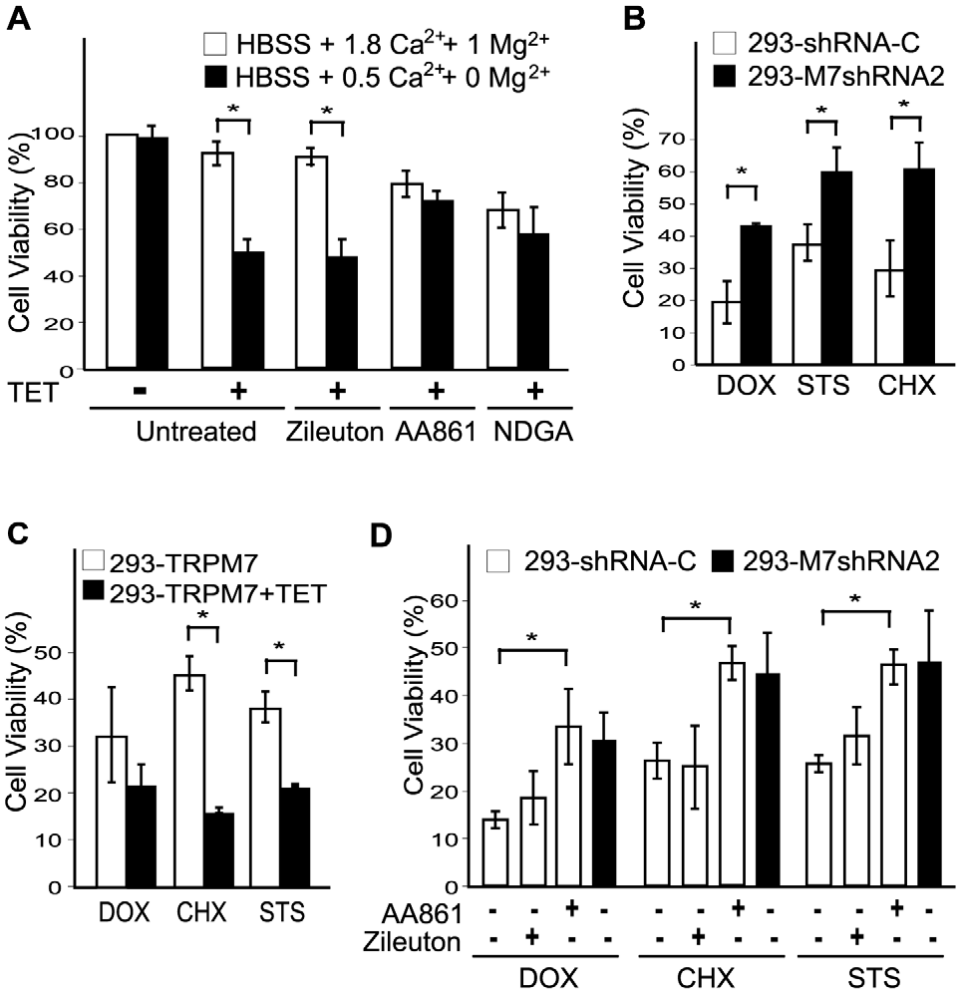
The effects of 5-LOX inhibitors on cell viability. (A) Overexpression of TRPM7 in HEK-293 cells cultured in low extracellular divalent cations (0.5 mM Ca^2+^ and 0 mM Mg^2+^) increased cell death compared to expressing cells grown in HBSS containing 1.8 mM Ca^2+^ and 1 mM Mg^2+^. Application of AA861 (10 µM) and NDGA (10 µM) but not zileuton (10 µM) increased the cell viability of TRPM7-expressing cells in response to reduced extracellular divalent cations. Cell viability was measured after 48 hours treatment by manual cell counting and trypan blue staining. (B) Knockdown of TRPM7 in HEK-293 cells rendered the cells more resistant to cell stress. Cell viability of TRPM7-knockdown 293-M7shRNA2 and the control 293-shRNA-C cell line in response to 48 hours treatment with the apoptotic stimulating agents doxorubicin (DOX) at 0.25 µM, cycloheximide (CHX) at 1 µg/ml, and staurosporine (STS) at 0.25 µM. Cell viability was measured using the MTT assay. (C) Overexpression of TRPM7 in 293-TRPM7 cells increased their sensitivity to cell death by DOX, STS, and CHX. 293-TRPM7 cells were treated with 0.0625 µM DOX, 1 µg/ml CHX, and 0.25 µM STS for 48 hours with or without pre-treatment with TET for 18 hours to induce protein expression. Cell viability was determined using the MTT assay. (D) Application of the 5-LOX inhibitor AA861 (10 µM), but not zileuton (10 µM), to 293-shRNA-C cells increased their resistance to cell death by DOX (0.25 µM), CHX (1 µg/ml) and STS (0.25 µM) to a level similar to 293-M7shRNA2 cells not treated with AA861. Cell viability was determined by manual cell counting and trypan blue staining. All data are presented as the mean ± standard deviation of three independent experiments. The asterisk indicates a significant difference in cell viability between two different treatments using Student's *t* test (*p*<0.05).

Knockdown of TRPM7 in isolated cortical neurons or in the hippocampus delayed neuronal death caused by oxygen glucose deprivation and brain ischemia, respectively [11], [36]. In a recent study, TRPM7 was additionally shown to increase cell death of neurons and HEK-293 cells cultured in low concentrations of extracellular divalent cations [36]. Interestingly, application of AA861 and NDGA reduced cell death of cells overexpressing TRPM7 that were cultured in low extracellular divalent cations (0 mM Mg^2+^/0.5 mM Ca^2+^) (Fig. 6A). Zileuton, which does not block the TRPM7 channel (Fig. 2G,H), did not reduce cell death under these conditions. MK886 was not employed in this assay, because it increased cell toxicity under these experimental conditions. While knockdown of TRPM7 in neurons is protective against oxygen glucose deprivation, it is unknown whether reducing TRPM7 expression in other cell types defends against other forms of cell stress stimuli. Previously, we created the 293-M7shRNA2 cell line expressing a shRNA targeting human TRPM7 under tetracycline (TET) control, in addition to the 293-shRNA-C cell line expressing a non-silencing control shRNA [10]. Western blotting and electrophysiological measurements demonstrated that expression of TRPM7 in 293-M7shRNA2 cells was reduced by approximately 80% compared to control cells [10]. Interestingly, depletion of TRPM7 in the 293-M7shRNA2 cell line rendered the cells more resistant to cell stress induced by several well known chemical apoptotic stimuli, including staurosporine, doxorubicin, and cycloheximide (Fig. 6B). Conversely, overexpression of TRPM7 in 293-TRPM7 cells increased the sensitivity of these cells to death by these same chemical agents (Fig. 6C). Having shown that knockdown of TRPM7 in HEK-293 cells protects against cell death by several forms of apoptotic stimuli, we next tested whether one of the TRPM7 channel blockers would similarly increase resistance to cell stress. Long-term treatment (48 hr) of HEK-293 cells with MK886 and NDGA caused cell toxicity, thereby precluding their use in this experiment. However, application of AA861 to 293-shRNA-C cells reduced cell death caused by exposure to staurosporine, doxorubicin, and cycloheximide to a level similar to that observed for the TRPM7-knockdown cell line (Fig. 6D). These data reveal the potential for TRPM7 channel blockers to be employed to reduce cell death under pathological conditions.

## Discussion

Stroke is the third leading cause of death in the United States as well as a major cause of disability [37]. Considerable efforts have been spent on developing treatments for stroke, but they have been met with limited success [11]. For example, the contribution of excitotoxicity mediated by glutamatergic NMDA receptors to ischemia-induced cell death is well appreciated, however, blockers of these receptors are not well tolerated and are only effective for a very short time following the onset of ischemia [11]. Thus, the identification of new targets for pharmacological intervention in stroke is urgently needed.

Two studies by the same group have highlighted the importance of TRPM7 to ischemic cell death. The earlier study demonstrated that knockdown of TRPM7 dramatically reduced neuronal cell death induced by oxygen glucose deprivation (OGD) using an *in vitro* model of ischemia [11]. This work was subsequently followed by experiments to demonstrate the protective effect of knockdown of TRPM7 by RNA interference following ischemia using an *in vivo* model [12]. More recently, a study by Inoue and coworkers revealed that TRPM7 is involved in Zn^2+^-induced injury of cultured mouse cortical neurons [38]. Collectively, these studies strongly suggest that TRPM7 may be an effective pharmacological target for stroke treatment; however, compounds that could potentially be used clinically against the channel have not been identified.

In this study we have identified the 5-LOX inhibitors NDGA, AA861, and MK886 as potent blockers of TRPM7 channel activity. The compounds were also effective at inhibiting TRPM7 channel function, as application of these molecules prevented TRPM7-induced cell rounding as well as cell death caused by low extracellular divalent cations or several forms of apoptotic stimuli. NDGA, AA861, and MK886 were originally identified by their capacity to inhibit 5-LOX [39], [40], [41], however, several lines of evidence suggest that these compounds block TRPM7 channel currents directly and independent of their inhibitory effects on 5-LOX enzymatic activity. Transfection of the dsiRNA targeting 5-LOX failed to lower TRPM7 whole cell currents compared to cells transfected with the control dsiRNA, although transfection of dsiRNAs targeting the 5-LOX partially interfered with TRPM7-mediated cell rounding. It has been reported that 5-LOX is involved in the regulation of cell adhesion, so the effects of the 5-LOX dsiRNAs on TRPM7-induced cell rounding are likely due to direct knockdown of 5-LOX expression [24]. In addition, we were unable to reverse AA861's blockade of TRPM7 channel activity by perfusion of the 5-LOX product 5-HPETE or its downstream metabolites into the extracellular bath solution. Likewise, inclusion of either 5-HPETE, LTD4, and LTB4 into the internal pipette solution did not prevent the inhibition of TRPM7 channel activity by AA861. Finally, the other two 5-LOX inhibitors, 5,6-DAA and zileuton, were ineffective in blocking TRPM7 currents. Collectively, these results strongly indicate that NDGA, AA861, and MK886 block TRPM7 channel currents independent of their actions on 5-LOX enzymatic activity.

NDGA, AA861, and MK886 did not alter TRPM7 protein expression or its concentration on the cell surface, leaving it unclear how these compounds may be interfering with TRPM7 channel activity. NDGA is a lipophilic reducing agent that blocks catalysis by reducing the active site iron in 5-LOX, whereas AA861 competes with binding of arachadonic acid to the enzyme [21], [42]. The structurally unrelated indole-containing MK886 is also lipophilic, blocking 5-LOX activity by binding to FLAP, a membrane protein that facilitates 5-lipoxygenase enzymatic activity by enhancing the delivery of arachidonic acid to 5-LOX [43]. Thus, the compounds may be blocking TRPM7 directly in the membrane or by interfering with binding of lipid to the channel.

Since NDGA, AA861, and MK886 effectively block the endogenous TRPM7 current, a reevaluation of the results of experimental studies employing these compounds is warranted. Administration of 5-LOX inhibitors has been shown to reduce tissue damage in rodent models of cerebral ischemia and myocardial ischemia-reperfusion injury [44], [45], [46], [47]. However, no significant difference in the infarct size between control and 5-LOX knockout mice was observed using either a heart or brain model of ischemic injury [48], [49]. As knockdown of the TRPM7 channel reduces the pathogenesis of brain ischemia, it is tempting to speculate that 5-LOX inhibitors achieve a portion of their cellular protective effects by blocking the TRPM7 channel. Indeed, the 5-LOX inhibitors AA861 and NDGA were effective in reversing TRPM7-induced cell death when cells are cultured in low extracellular divalent cations. In addition, both knockdown of TRPM7 and application of AA861 were effective in reducing cell death caused by apoptotic stimuli. We conclude that NDGA, AA861, and MK886 are effective blockers of TRPM7 channel activity independent of their actions on 5-LOX. These compounds will be valuable reagents for identifying and characterizing native TRPM7 currents, as well as for blocking the physiological and pathological functions of the channel *in vivo*.

## Materials and Methods

### Reagents

Indomethacin, a specific COX inhibitor, nordihydroguaiaretic acid (NDGA), a lipoxygenase inhibitor, and staurosporine were from Calbiochem/EMD Biosciences (San Diego, CA). Baicalein, a selective inhibitor of 12-LOX, PD146176, a selective inhibitor of 15-LOX, two kinds of 5-LOX specific inhibitors, 2-(12-Hydroxydodeca-5,10-diynyl)-3,5,6-trimethyl-1,4-benzoquinone (AA861) and 5,6-dehydro-arachidonic acid (5,6-DAA), doxorubicin and cycloheximide were from Sigma (St. Louis, MI). 3-[1-(4-chlorobenzyl)-3-t-butyl-thio-5-isopropylindol-2-yl]-2,2-dimethylpropanoic acid (MK886), a specific inhibitor of FLAP, and two kinds of downstream metabolites from 5-hydroperoxyeicosatetraenoic acid (5-HPETE), leukotriene B4 and leukotriene D4, were from Cayman (Ann Arbor, MI). 5-HPETE, and arachidonic acid, the product and substrate of 5-LOX respectively, were from Biomol (Plymouth, PA). (*RS*)-*N*-[1-(1-benzothien-2-yl)ethyl]-*N*-hydroxyurea (zileuton), the inhibitor of 5-LOX, was from Tocris (Ellisville, MO). All other chemicals were from Sigma (St. Louis, MI) unless otherwise indicated.

### Cell Lines

A description and characterization of the 293-TRPM7 cell line expressing hemagglutinin (HA) tagged murine TRPM7 (GenBank Accession # **AF376052** ), of the 293-M7shRNA2 cells expressing a shRNA targeting human TRPM7 under tetracycline control, and of the 293-shRNA-C cells expressing a non-silencing control shRNA was previously described [10]. The Flp-In T-Rex 293 cell line (HEK-TR) used to construct 293-M7shRNA2, 293-shRNA-C, and 293-TRPM7 cell lines was from Invitrogen (Carlsbad, CA). The overexpression of HA-tagged TRPM7 in 293-TRPM7 cells was induced by the addition of tetracycline (1 µg/ml) to the growth medium.

### Cell Rounding Assay

Phase-contrast images of 293-TRPM7 cells were obtained with a phase-contrast 10X UPlanFI objective using an Olympus IX70 microscope equipped with an environmental chamber at a temperature of 37°C. Three random fields were selected in each treatment, and every treatment was repeated in three independent experiments. Changes in cell morphology were scored manually employing the following criteria: Cells that had a fully-rounded cell body with no membrane extension processes were given one point. Partially-rounded cells with one or two membrane extension processes were assigned half-a-point. Non-rounded cells having three or four membrane extension processes and with a cell morphology similar to wildtype HEK-293 cells, were given zero points. The IC_50_ was determined by plotting the logarithm of degree of cell rounding versus the logarithm of the concentration of drug used and fitting to a modified version of the Hill equation. A χ^2^ test was used to test differences in cell rounding.

### Knockdown of 5-lipoxygenase in 293-TRPM7 Cells

The Dicer substrate RNA interferences (dsiRNAs) used to knockdown 5-LOX were from Integrated DNA Technologies (Coralville, IA). The following oligoribonucleotide pairs were used to create the dsiRNAs: dsiLOX5-1; 5′GCA ACA CCG ACG UAA AGA ACU GGA A-3′ and 5′-UUC CAG UUC UUU ACG UCG GUG UUG CUU-3′ and for dsiLOX5-2; 5′-GGU AGA CAU CUA CUA CGA GGG CGAC-3′ and 5′-GUC GCC CUC GUA GUA GAU GUC UAC CAC-3′. A nonsilencing sequence tagged with Cy3 (5′Cy3-TCC UUC CUC UCU UUC UCU CCC UUG UGA-3′ and 5′Cy3-TCA CAA GGG AGA GAA AGA GAG GAA GGA) was used to make dsiCT. 293-TRPM7 cells were transfected with 10 nM of dsiRNA (dsiCT or dsiLOX5) using Lipofectamine 2000 (Invitrogen, Carlsbad, CA). To detect 5-LOX, cells were lysed in ice-cold radioimmunoprecipitation assay (RIPA) buffer (50 mM Tris (pH 7.4), 150 mM NaCl, 1 mM ethylenediaminetetraacetic acid, 1% IGEPAL CA-630, 0.5% (w/v) deoxycholate, 0.1% (w/v) SDS, and 10 mM iodoacetamide) containing protease inhibitors (Roche Applied Science, Indianapolis, IN). 5-LOX was resolved by sodium dodecyl sulfate polyacrylamide gel electrophoresis (SDS-PAGE) and western blotting using the polyclonal antibody (N-19) against 5-LOX (Santa Cruz Biotechnology, Santa Cruz, CA). The SuperSignal West Dura Maximum Sensitive Substrate (Pierce, Rockford, IL) was used for immunochemiluminescence detection.

### Detection of Total and Cell Surface TRPM7 Protein Expression

293-TRPM7 cells were washed three times with ice-cold PBS. Intact cells were incubated with 1 mg/ml sulfo-NHS-LC-biotin (Pierce, Rockford, IL) for 30 min at 4°C. Unreacted biotin was quenched by 10 mM glycine in PBS three times. Cells were lysed in ice-cold RIPA buffer containing protease inhibitors. Cell lysates were incubated with anti-HA affinity matrix (Sigma) at 4°C overnight. The immunoprecipitates were washed extensively and analyzed by SDS-PAGE and western blotting with avidin-horseradish peroxidase (Bio-Rad, Hercules, CA) for detecting surface expression of TRPM7 and a monoclonal HA antibody (Roche Applied Science) for detecting TRPM7. The SuperSignal West Dura Maximum Sensitive Substrate (Pierce, Rockford, IL) was used for immunochemiluminescence detection.

### Electrophysiological Recordings

The voltage-clamp technique used to evaluate the whole-cell currents of TRPM7 expressed in HEK-293 cells was previously described [10]. Briefly, whole-cell current recordings of TRPM7-expressing cells were elicited by voltage stimuli lasting 250 ms delivered every 1 second using voltage ramps from −120 to +100 mV. Data was digitized at 2 or 5 kHz and digitally filtered off-line at 1 kHz. The internal pipette solution for macroscopic current recordings contained (in mM) 145 Cs-methanesulfonate, 8 NaCl, 10 EGTA, and 10 4-(2-hydroxyethyl)-1-piperazineethanesulfonic acid (HEPES), pH adjusted to 7.2 with CsOH. The free Mg^2+^ concentration in the pipette was <1 nM. The extracellular solution for whole-cell recordings contained (in mM) 140 NaCl, 5 KCl, 2 CaCl_2_, 20 HEPES, and 10 glucose, pH adjusted to 7.4 with NaOH. Extracellular solution was perfused through a fast-perfusion system which can exchange external solutions within 3 s. Unless otherwise stated, each compound was included in the external solution and applied to the cells after TRPM7 current reached steady state. Compounds were washed out once the maximal effect was observed.

### Cell Viability Measurements

The assay of the effects of low extracellular divalent cations on the cell viability of 293-TRM7 expressing cells was performed as described with the following modifications [36]. Briefly, 293-TRPM7 cells were cultured on poly-L-lysine coated plates in Dulbecco's Modified Eagle Medium (DMEM) with 10% fetal bovine serum (FBS). After 20 hr with or without tetracycline induction, cells were washed twice with Hanks' balanced salt solution (HBSS) (121 mM NaCl, 5 mM KCl, 20 mM D-glucose, 20 mM Hepes, and 1 mM Na-pyruvate). Cells were incubated in HBSS with normal divalent cations (1 mM MgCl_2_, 1.8 mM CaCl_2_) or with reduced extracellular divalent cations (0 mM MgCl_2_, 0.5 mM CaCl_2_) and treated with 10 µM of the following inhibitors, Zileuton, AA861 and NDGA, at 37°C for 48 hr. Cells were then trypsinized and cell viability was determined using the trypan blue exclusion assay.

To assess the resistance of cells to apoptotic stimuli cells were treated with doxorubicin (DOX), staurosporine (STS), or cycloheximide (CHX) for 48 hrs to induce cell death. Viability was determined by MTT assay or manual cell counting by trypan blue exclusion assay. For the MTT assay, cells were incubated with 0.1 mg/ml 3-(4,5-Dimethylthiazol-2-yl)-2,5-diphenyltetrazolium bromide (MTT) for 4 hours and then dissolved in 100 µl dimethyl sulfoxide. The absorbance at 570 nm was measured and normalized to untreated cells.

## Supporting Information

Figure S1 The effects of higher concentrations of 5-LOX inhibitors on TRPM7 channel activity. Application of the 5-LOX inhibitors AA861 (A) and NDGA (C) to 293-TRPM7 expressing cells decreased TRPM7 current amplitude over time (+100 mV). Representative traces showing the TRPM7 current-voltage relationship before and after application of AA861 (B) and NDGA (D). The above experiments were performed a minimum of 3 to 5 times with similar results.
(0.32 MB TIF)

Figure S2 Characterization of the 5-LOX dsiRNAs. (A) A western blot demonstrating that cotransfection of cDNA encoding GFP-5-LOX with dsiRNA targeting 5-LOX (dsiLOX5-1 & dsiLOX5-2) reduced expression of GFP-5-LOX compared to cells transfected with GFP-5-LOX alone (-) or with GFP-5-LOX and the control dsiRNA (dsiCT). A western blot of β-actin is shown to demonstrate equal loading of the samples. (B) Western blot showing that cotransfection of dsiLOX5-1 and dsiLOX5-2 with GFP-15-LOX-2 did not reduce expression of GFP-15-LOX-2 compared to cells transfected with GFP-15-LOX-2 alone or with GFP-15-LOX-2 and the control dsiRNA (dsiCT). A western blot of β-actin is shown to demonstrate equal loading of the samples.
(0.24 MB TIF)

Figure S3 The 5-LOX product 5-HPETE and its metabolites LTB4 and LTD4 do not reverse inhibition of TRPM7 current by AA861. Application of the 5-LOX inhibitor AA861 to 293-TRPM7 expressing cells dramatically reduced TRPM7 current amplitudes (+100 mV). Coadministration of the 5-LOX product 5-HPETE (A) as well as its metabolites LTB4 (C) and LTD4 (E) to the external solution did not restore TRPM7 current amplitudes over time. Similarly, inclusion of 5-HPETE (B), LTB4 (D), and LTD4 (F) in the internal pipette solution did not prevent inhibition of TRPM7 channel activity by AA861. The above experiments were performed a minimum of 10 times with similar results.
(0.27 MB TIF)

Figure S4 Effects of the 5-LOX inhibitor 5,6-DAA and arachidonic acid on TRPM7 channel activity. (A) Application of the 5-LOX inhibitor 5,6-DAA (5 µM) to 293-TRPM7 expressing cells had no effect on TRPM7 current amplitudes (+100 mV, n = 5). 1.5% ethanol (EtOH) was employed as a vehicle control and caused a very small decrease in the current amplitude. (B) Representative traces showing the TRPM7 current-voltage relationship before and after application of 5,6-DAA and 1.5% ethanol (EtOH). (C) Application of arachidonic acid (10 µM) to 293-TRPM7 expressing cells had no effect on TRPM7 current amplitudes (+100 mV, n = 6). (D) Representative traces showing the TRPM7 current-voltage relationship before and after application of arachidonic acid.
(0.39 MB TIF)
