# Supplementary figures and images for: Blockade of TRPM7 Channel Activity and Cell Death by Inhibitors of 5-Lipoxygenase
Source: PLoS One. 2010 Jun 17;5(6):e11161. doi: 10.1371/journal.pone.0011161 (PMC2887440; doi:10.1371/journal.pone.0011161)

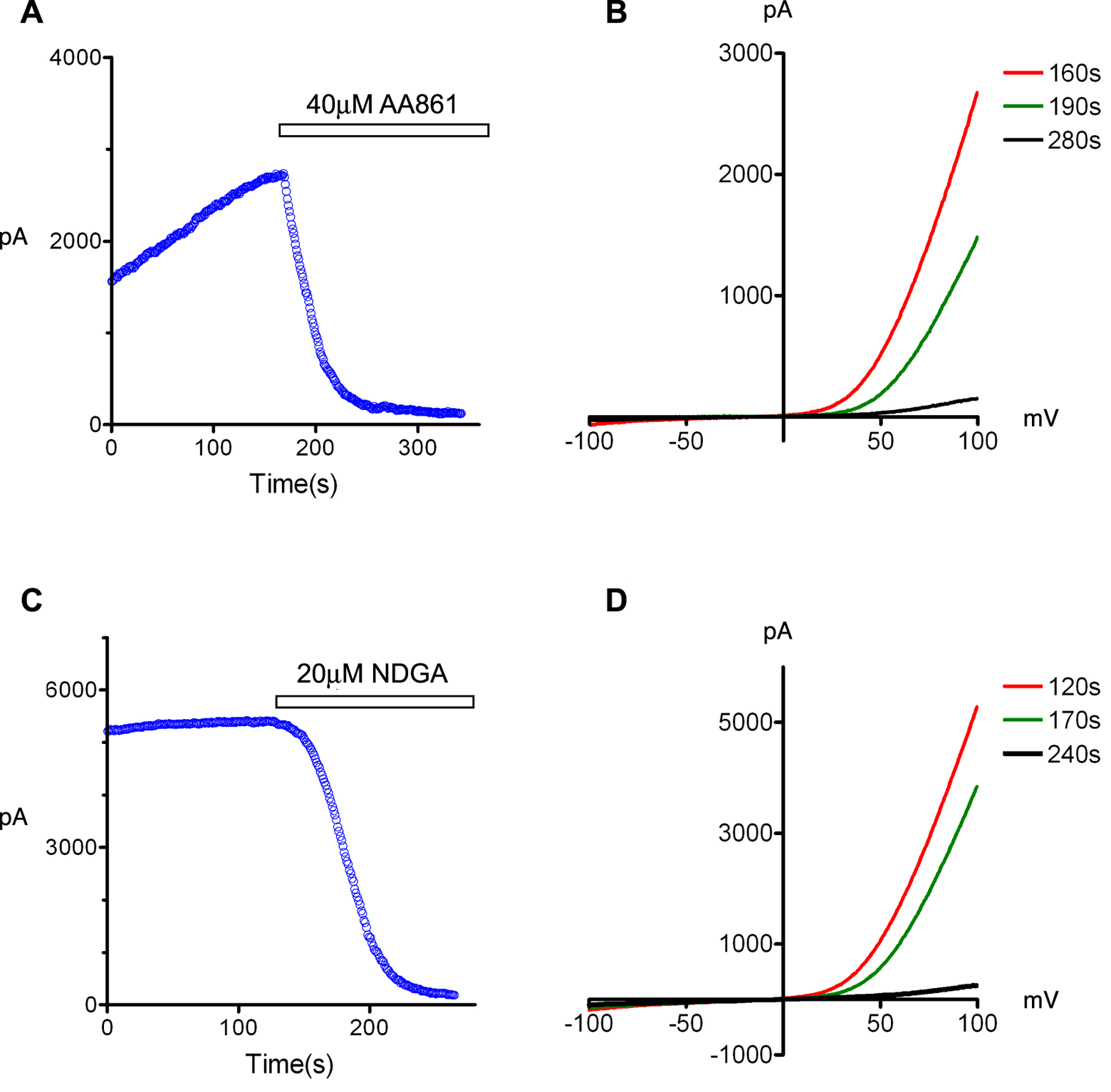

Supplement: Figure S1 — The effects of higher concentrations of 5-LOX inhibitors on TRPM7 channel activity. Application of the 5-LOX inhibitors AA861 (A) and NDGA (C) to 293-TRPM7 expressing cells decreased TRPM7 current amplitude over time (+100 mV). Representative traces showing the TRPM7 current-voltage relationship before and after application of AA861 (B) and NDGA (D). The above experiments were performed a minimum of 3 to 5 times with similar results. (0.32 MB TIF) [file pone.0011161.s001.tif]

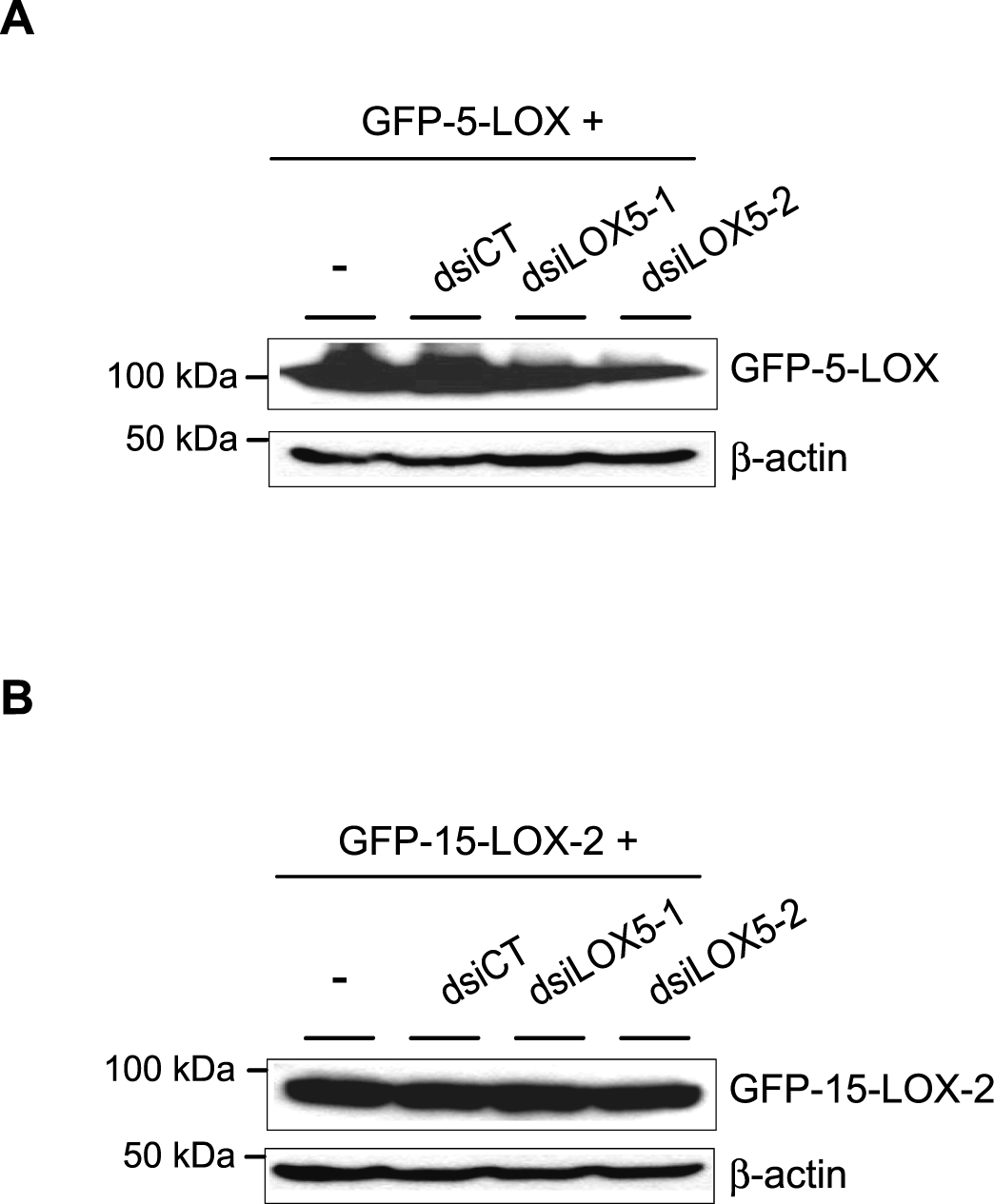

Supplement: Figure S2 — Characterization of the 5-LOX dsiRNAs. (A) A western blot demonstrating that cotransfection of cDNA encoding GFP-5-LOX with dsiRNA targeting 5-LOX (dsiLOX5-1 & dsiLOX5-2) reduced expression of GFP-5-LOX compared to cells transfected with GFP-5-LOX alone (-) or with GFP-5-LOX and the control dsiRNA (dsiCT). A western blot of β-actin is shown to demonstrate equal loading of the samples. (B) Western blot showing that cotransfection of dsiLOX5-1 and dsiLOX5-2 with GFP-15-LOX-2 did not reduce expression of GFP-15-LOX-2 compared to cells transfected with GFP-15-LOX-2 alone or with GFP-15-LOX-2 and the control dsiRNA (dsiCT). A western blot of β-actin is shown to demonstrate equal loading of the samples. (0.24 MB TIF) [file pone.0011161.s002.tif]

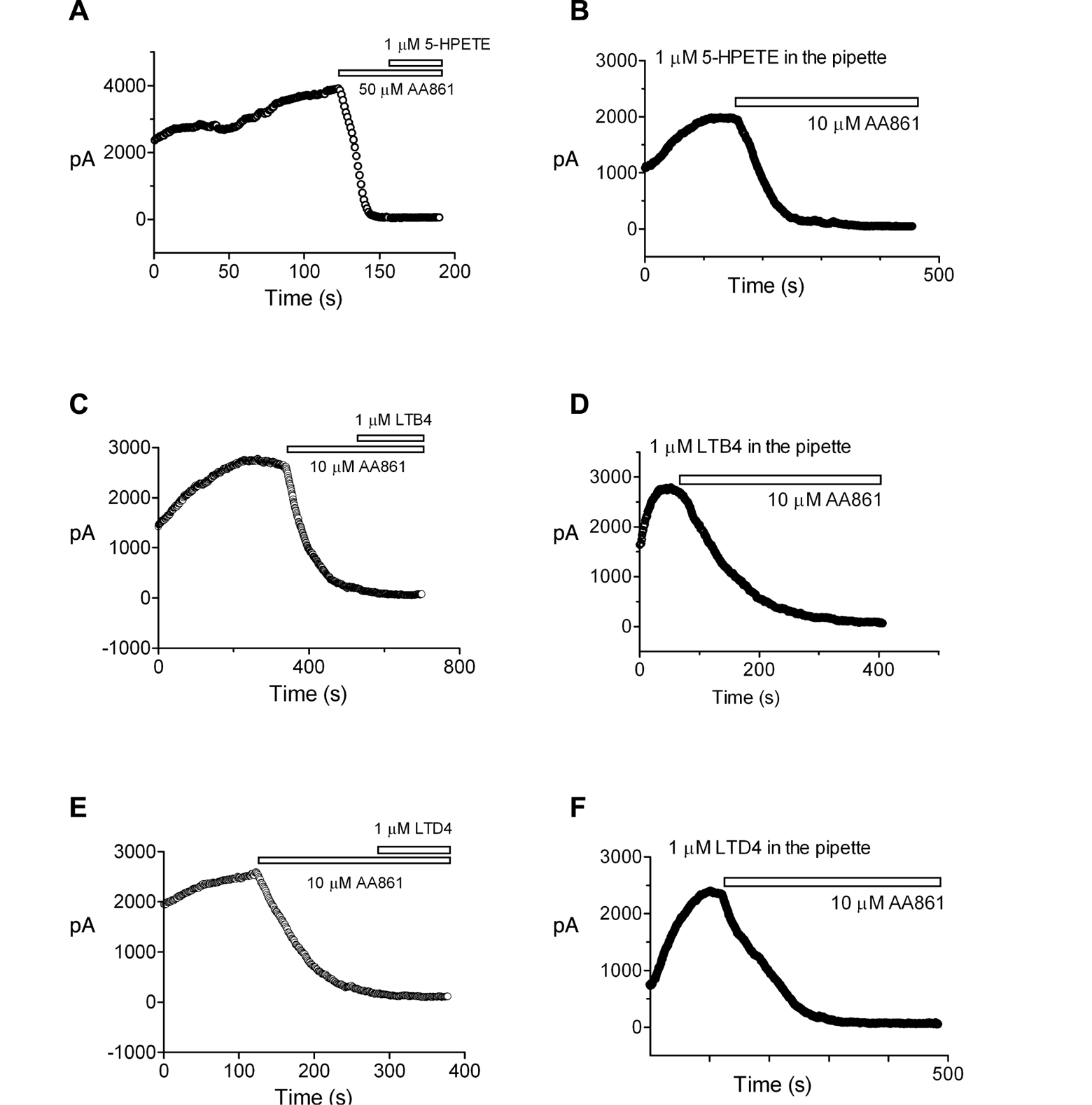

Supplement: Figure S3 — The 5-LOX product 5-HPETE and its metabolites LTB4 and LTD4 do not reverse inhibition of TRPM7 current by AA861. Application of the 5-LOX inhibitor AA861 to 293-TRPM7 expressing cells dramatically reduced TRPM7 current amplitudes (+100 mV). Coadministration of the 5-LOX product 5-HPETE (A) as well as its metabolites LTB4 (C) and LTD4 (E) to the external solution did not restore TRPM7 current amplitudes over time. Similarly, inclusion of 5-HPETE (B), LTB4 (D), and LTD4 (F) in the internal pipette solution did not prevent inhibition of TRPM7 channel activity by AA861. The above experiments were performed a minimum of 10 times with similar results. (0.27 MB TIF) [file pone.0011161.s003.tif]

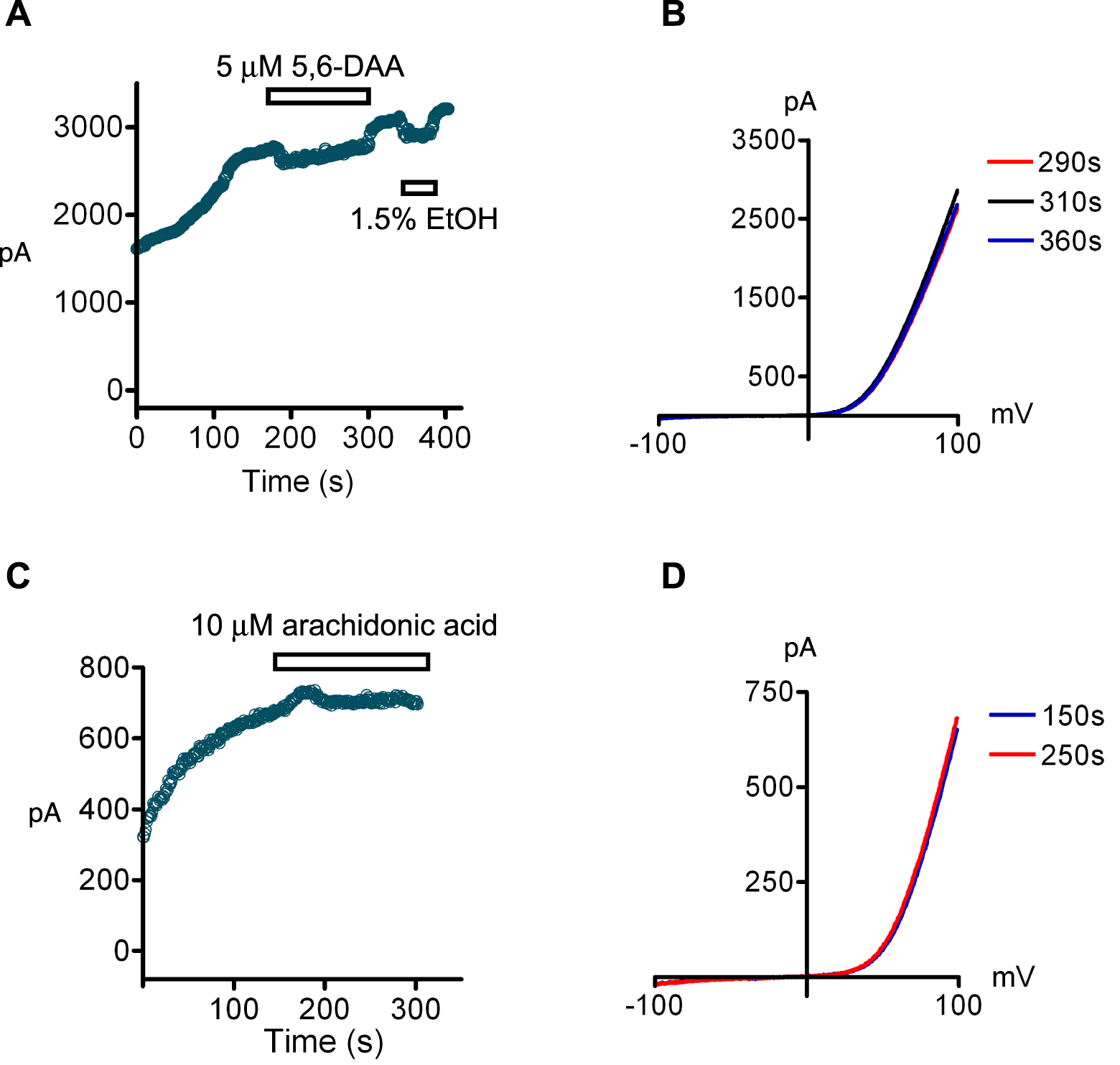

Supplement: Figure S4 — Effects of the 5-LOX inhibitor 5,6-DAA and arachidonic acid on TRPM7 channel activity. (A) Application of the 5-LOX inhibitor 5,6-DAA (5 µM) to 293-TRPM7 expressing cells had no effect on TRPM7 current amplitudes (+100 mV, n = 5). 1.5% ethanol (EtOH) was employed as a vehicle control and caused a very small decrease in the current amplitude. (B) Representative traces showing the TRPM7 current-voltage relationship before and after application of 5,6-DAA and 1.5% ethanol (EtOH). (C) Application of arachidonic acid (10 µM) to 293-TRPM7 expressing cells had no effect on TRPM7 current amplitudes (+100 mV, n = 6). (D) Representative traces showing the TRPM7 current-voltage relationship before and after application of arachidonic acid. (0.39 MB TIF) [file pone.0011161.s004.tif]
